# Quality of life of men with AIDS and the model of social determinants of health^1^

**DOI:** 10.1590/0104-1169.0120.2541

**Published:** 2015

**Authors:** Gilmara Holanda da Cunha, Maria Luciana Teles Fiuza, Elucir Gir, Priscila de Souza Aquino, Ana Karina Bezerra Pinheiro, Marli Teresinha Gimeniz Galvão

**Affiliations:** 2 PhD, Professor, Departamento de Enfermagem, Universidade Federal do Ceará, Fortaleza, CE, Brazil; 3 MSc, RN, Hospital Universitário Walter Cantídio, Fortaleza, CE, Brazil; 4 PhD, Full Professor, Escola de Enfermagem de Ribeirão Preto, Universidade de São Paulo, WHO Collaborating Centre for Nursing Research Development, Ribeirão Preto, SP, Brazil; 5 PhD, Associate Professor, Departamento de Enfermagem, Universidade Federal do Ceará, Fortaleza, CE, Brazil

**Keywords:** HIV, Acquired Immunodeficiency Syndrome, Health Promotion, Quality of Life, Nursing

## Abstract

**OBJECTIVE:**

to analyze the quality of life (QoL) of men with AIDS from the perspective of the model of social determinants of health (MSDH).

**METHOD:**

cross-sectional study conducted in an outpatient infectious diseases clinic from a Brazilian university hospital over the course of one year with a sample of 138 patients. A form based on the MSDH was used to collect sociodemographic data addressing individual, proximal, intermediate determinants and the influence of social networks together with an instrument used to assess the QoL of people with HIV/AIDS. The project was approved by the Institutional Review Board (Protocol No. 040.06.12).

**RESULTS:**

according to MSDH, most men with AIDS were between 30 and 49 years old (68.1%), mixed race (59.4%), heterosexual (46.4%), single (64.5%), Catholic (68.8%), had a bachelor's degree (39.2%), had no children (61.6%), and had a formal job (71.0%). The perception of QoL in the physical, level of independence, environment, and spirituality domains was intermediate, while QoL was perceived to be superior in the domains of psychological and social relationship. A perception of lower QoL was presented by homosexual (p=0.037) and married men (p=0.077), and those with income below one times the minimum wage (p=0.042). A perception of greater QoL was presented by those without a religion (p=0.005), living with a partner (p=0.049), and those who had a formal job (p=0.045).

**CONCLUSION:**

social determinants influence the QoL of men with AIDS.

## Introduction

The Human Immunodeficiency Virus (HIV) infection is currently a health problem due to its pandemic nature and severity. A total of 686,478 cases of AIDS were reported to the Notifiable Diseases Information System (SINAN) from 1980 to December 2013; 445,197 of which refer to men and 241,223 to women^(^ 1 ^)^. The epidemic is currently stable in Brazil and concentrated in vulnerable population groups, but male adults are still the most frequently affected^(^ 1 ^-^ 2 ^)^.

The availability of antiretroviral treatment (ART) in Brazil since 1996 has enabled a reduction in both morbidity and mortality caused by HIV. Brazil was the first developing country to adopt a public policy for patients to have access to ART and is recognized worldwide for providing a program that has achieved good response against the HIV/AIDS epidemic^(^ 3 ^)^.

Even though there is a national program and very well-organized to combat sexually transmitted diseases (STD) and AIDS, one should consider that economic and political aspects have an essential meaning to each population in which AIDS has been disseminated. Most cases of AIDS are located in poor countries, suggesting the need to analyze the social determinants of health in these populations^(^ 4 ^)^.

Because ART has enabled increased survival rates of people with HIV/AIDS, characterizing it as a chronic disease, the health care provided to these individuals gains greater importance for these individuals who require unique care procedures to maintain their quality of life (QoL)

The World Health Organization (WHO) developed, within a multi-center collaborative study, an instrument to assess QoL from an international and cross-cultural perspective. This instrument is called the World Health Organization Quality of Life (WHOQOL-100), which originated a specific instrument for people living with HIV/AIDS, WHOQOL-HIV^(^ 5 ^)^ that has been already validated in Brazil^(^ 6 ^)^. QoL is a perception of individuals in regard to their position in life in the context of the culture and value system in which they live and in relation to their objectives, expectations, standards and concerns^(^ 7 ^)^. Studies show that the QoL of people with HIV/AIDS is compromised due to discrimination and the unfavorable socioeconomic conditions in which they live, which may determine increased rates of mortality^(^ 8 ^-^ 10 ^)^.

Given the previous discussion and with the intent to contribute to the quality of healthcare provided to people living with HIV/AIDS, considering that most individuals affected by HIV infection are male and that social determinants impacting this process, we proposed this study to analyze the QoL of men with AIDS using the WHOQOL-HIV bref, with the Model of Social Determinants of Health (MSDH) as theoretical framework.

## Theoretical framework

The social determinants of health are the social conditions in which people live and that affect people's health. There are different models of social determinants of health but the National Commission on the Social Determinants of Health chose Dahlgren and Whitehead's model to be used in Brazil due to its simplicity and clear graphic representation of social determinants^(^ 11 ^)^.

In this model^(^ 12 ^)^, the social determinants of health are displayed in concentric layers, where individuals are in the center of the model: layer 1 (individual determinants: age, sex, genetic inheritance); layer 2 (proximal determinants: individual behavior and lifestyle); layer 3 (influence of social networks); layer 4 (intermediary determinates: living conditions, work, food, access to basic environments and services such as healthcare, education, sanitation, housing); layer 5 (distal or macro-determinants: society's economic, cultural and environmental conditions, including supranational determinants, such as globalization).

## Method

This cross-sectional study with quantitative approach was conducted in an infectious disease outpatient clinic in a Brazilian university hospital that provides care to adults with HIV/AIDS. This hospital has an outpatient service, inpatient wards and an intensive care unit and also provides laboratorial and radiological exams, ART and condoms.

Data were collected over the course of one year. In order to meet the study's objectives, the sample size was determined to estimate the mean of the scores obtained in the domains assessing the QoL of people living with HIV/AIDS, the WHOQOL-HIV bref^(^ 6 ^)^, with a 95% confidence level that the estimation error would not surpass 3%, considering that the mean of these scores, according to prior studies^(^ 13 ^-^ 14 ^)^, is about 13.7, with a standard error of 3.8, and also that there were 235 male patients receiving care from the outpatient clinic up to the end of 2001. For that, the following expression was used:



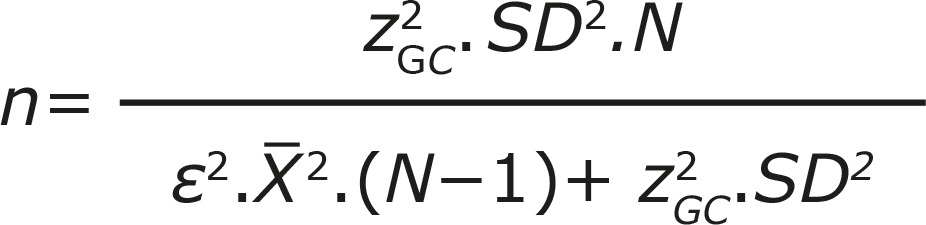



In this formula, z_GC_ is equal to the z-statistic (1.96), standard deviation (SD), X̅ and ε correspond to mean and acceptable error (0.03), respectively. Hence, a sample of 138 patients was computed.

Inclusion criteria were: being a man and having AIDS; being older than 28 years of age; being in outpatient follow-up; using ART; and signing a free and informed consent form. Exclusion criteria were having access to ART at the hospital but with follow-up performed in a private facility; suffering from mental disease or any other condition that would impede the individual from answering the study's forms. The participants were invited to participate in the study when they attended the outpatient consultations. Two forms were applied through interviews in a private room. 

A form was developed in accordance with MSDH^(12) ^to address the sociodemographic characterization of people with HIV/AIDS and was arranged into four layers 1- identification, age, race; 2 - category of exposure, marital status, religion, schooling; 3 - number of children, number of household members, and whether the individual lives with a partner; 4 - occupation and *per capita *income (times minimum wage). The form was based on the MSDH layers 1, 2, 3 and 4, while layer 5 was used in the discussion of findings because it refers to the distal or macro-determinants involving economic, cultural, and environmental conditions of society, current policies, supranational determinants and globalization and does not directly refer to the personal characteristics of the study's participants. The form was validated after being applied to 20 male individuals with AIDS, who did not compose part of the sample.

The other form was an instrument used to assess the QoL of people with HIV/AIDS, the World Health Organization Quality of Life instrument - HIV/AIDS module (WHOQOL-HIV), the Portuguese version of which has already been validated in Brazil^(^ 6 ^)^. The full version includes 120 questions (WHOQOL-HIV), so the brief version (WHOQOL-HIV bref) was used instead. It assesses the generic QoL of people with HIV/AIDS. WHOQOL-HIV bref has 31 items measuring QoL distributed into six domains: I. Physical (pain, discomfort, energy, fatigue, sleep, rest); II. Psychological (positive and negative feelings, cognition, self-esteem, body image, appearance); III. Level of Independence (mobility, activities of daily living, dependence on medication and treatment, work capacity); IV. Social Relationships (personal relationships, social support, sexual activity); V. Environment (physical safety and security, housing, finance, health and social care access, opportunities for acquiring new information and skills, leisure, physical environment, transportation); VI. Spirituality/religion/personal beliefs (forgiveness, blame, concerns about the future, death and dying). The questions are scored on a 5-point Likert scale containing 1 (not at all), 2 (a little), 3 (a moderate amount), 4 (very much) and 5 (an extreme amount), such that 1 indicates negative perceptions and 5 indicates positive perceptions. The scores obtained in each domain are scaled in a positive direction, where higher scores indicate better QoL. For the items pain, discomfort, negative feelings, dependence on medication, death and dying, scores are not scaled in a positive direction and higher scores do not represent better QoL and need to be inverted. Scores range from 4 to 20 points, which reflect worst and best QoL, respectively^(^ 6 ^)^.

The classification of previous studies addressing people living with HIV/AIDS was used to characterize the level of perception of QoL in each domain in which the scores translated, as follows: between 4 and 9.9 indicate a perception of low QoL, from 10 to 14.9 indicate a perception of intermediate QoL, and scores from 15 to 20 represent a perception of superior QoL^(^ 13 ^-^ 14 ^)^.

Data were organized in Microsoft Excel 2007^(r)^ and processed by the Statistical Package for Social Sciences (SPSS) version 18^(r)^. Absolute and relative frequencies, mean, standard deviation, median, minimum and maximum values were used in the descriptive statistics. Analysis of variance (ANOVA) was used to compare the means of the WHOQOL-HIV bref's domains with the variables of interest and Tukey's test and the T test were also applied for the intragroup analyses. Level of significance was established at 5% and considered statistically significant when *p* < 0.05.

The study project was approved by the Institutional Review Board on July 30, 2012 in accordance with Resolution No. 196/96, National Council of Health (Protocol No. 040.06.12). The participants signed free and informed consent forms and anonymity was ensured; the study's data were used only for scientific purposes.

## Results

Table 1 presents the sociodemographic characterization of men with AIDS according to MSDH's layers 1, 2, 3 and 4. The average age was 40 years old, with minimum and maximum values of 20 and 78 years, respectively. Individuals aged from 30 to 49 years old (68.1%) and of mixed race (59.4%) were in the majority. In regard to the category of exposure, heterosexual individuals predominated (46.4%). Most individuals were single during the time of the study (64.5%), reported being Catholic (68.8%), and had a bachelor's degree (39.2%).

**Table 1 -:** Distribution of men with AIDS according to layers 1, 2, 3 and 4 from the Model of Social Determinants of Health (N=138). Fortaleza, CE, Brazil, 2013

Layers according to the Model of Social Determinants of Health			n (%)
1. Individual determinants			
	Age group (in years)*		
		≤ 29 years old	17 (12.3)
		30-49 years old	94 (68.1)
		≥ 50 years old	27 (19.6)
	Race		
		Caucasian	42 (30.4)
		Afro descendant	14 (10.2)
		Mixed	82 (59.4)
2. Proximal determinants			
	Category of exposure		
		Heterosexual	64 (46.4)
		Homosexual	55 (39.9)
		Bisexual	19 (13.7)
	Marital status		
		Single	89 (64.5)
		Married/stable union	39 (28.2)
		Others (divorced, widowed)	10 (7.3)
	Religion		
		Catholic	95 (68.8)
		No religion	10 (7.3)
		Other	33 (23.9)
	Schooling^†^		
		Illiterate	7 (5.1)
		Middle School	39 (28.2)
		High school	38 (27.5)
		Bachelor’s degree	54 (39.2)
3. Influence of social networks			
	Number of children^‡^		
		No children	85 (61.6)
		1 – 2	37 (26.8)
		≥ 3	16 (11.6)
	Number of household members§		
		1 – 2	57 (41.3)
		3 – 5	65 (47.1)
		≥ 6	16 (11.6)
	Lives with a partner		
		Yes	80 (58.0)
		No	58 (42.0)
4. Intermediate determinant			
	Occupation		
		Has a formal occupation	98 (71.0)
		No formal occupational	21 (15.2)
		Other	19 (13.8)
	Income *per capita* ^||^		
		< 1 times the minimum income	5 (3.6)
		1 - 2 times the minimum income	53 (38.4)
		> 2 times the minimum income	80 (58.0)

Mean ± standard deviation:

* Age (40 ± 10.77);

† Years of schooling (11± 4.99);

‡ Number of children (1 ± 1.52);

§ Number of household members (3 ± 1.82);

|| Minimum wage in 2013 in Brazil at the time of the study: R$ 678.00.

Most men had no children (61.6%) and only 11.6% reported more than six children, with nine being the highest number of children reported. Most lived together with three to five people (47.1%), had a formal job at the time of the study (71.0%), and 58.0% reported a *per capita* monthly income more than two times the minimum wage.

Table 2 shows the distribution of parameters regarding the WHOQOL-HIV bref's domains. Perception of QoL regarding the domains: physical, level of independence, environment, and spirituality was intermediate, while the perception of QoL for the psychological and social relationships domains were that QoL was superior.

**Table 2 -:** Distribution of scores regarding the domains of the WHOQOL-HIV bref applied to men with AIDS in outpatient follow-up (N=138). Fortaleza, CE, Brazil, 2013

WHOQOL-HIV Bref’s domains	Mean ± Standard deviation	Median	Minimum value	Maximum value
I. Physical	14.68 ± 3.28	15.00	7	20
II. Psychological	15.32 ± 2.51	15.20	6.4	19.2
III. Level of independence	13.86 ± 2.87	14.00	5	20
IV. Social relationships	15.50 ± 2.80	16	7	20
V. Environment	14.40 ± 2.12	14.50	9	18.50
VI. Spirituality	14.09 ± 3.88	13.50	7	20

WHOQOL-HIV bref: World Health Organization Quality of Life instrument-HIV bref

The analysis of association between the scores of the WHOQOL-HIV bref's scores and the MSDH's layers 1 and 2 presented statistical significance in some situations. The mean scores of homosexual participants for the domain VI were lower than that of heterosexual men, showing a perception of lower QoL concerning spirituality (p=0.037). Married men obtained lower scores in domain V (environment) in comparison to single men (p=0.077). Those who did not report a religion scored higher than the Catholic participants in the following domains: I (p=0.005), II (p=0.017) and III (p=0.038) (Table 3).

**Table 3 -:** Distribution of men with AIDS according to layers 1 and 2 of the Model of Social Determinants of Health and scores obtained for the domains of the WHOQOL-HIV bref (N=138). Fortaleza, CE, Brazil, 2013

Social Determination Model of Health			Scores obtained for the WHOQOL-HIV bref domains (Mean ± Standard Deviation)					
			I	II	III	IV	V	VI
Layer 1								
	Age group (in years)							
		≤ 29	15.5 ± 3.3	15.2 ± 2.8	14.2 ± 2.3	16.4 ± 2.3	14.5 ± 2.0	13.4 ±4.4
		30-49	15.0 ± 3.3	15.5 ± 2.5	14.0 ± 2.8	15.4 ± 2.9	14.5 ± 2.1	14.2 ± 3.9
		≥ 50	13.5 ± 2.7	14.9 ± 2.2	13.0 ± 3.1	15.4 ± 2.3	14.1 ± 2.0	14.0 ± 3.5
		P value	0.223	0.159	0.676	0.739	0.788	0.211
	Race (self-reported)							
		Caucasian	14.5 ± 3.0	14.9 ± 2.9	13.7 ± 2.7	15.2 ± 3.0	14.5 ± 1.9	13.1 ± 4.3
		Afro descendant	15.2 ± 3.2	15.4 ± 2.1	14.3 ± 3.1	15.5 ± 2.3	13.9 ± 2.4	14.7 ± 2.9
		Mixed	14.8 ± 3.3	15.5 ± 2.3	13.7 ± 2.9	15.7 ± 2.7	14.4 ± 2.1	14.4 ± 3.8
		P value	0.790	0.427	0.801	0.847	0.621	0.241
Layer 2								
	Sexual Orientation							
		Heterosexual	14.4 ± 3.2	15.6 ± 2.0	13.5 ± 3.0	15.5 ± 2.7	14.0 ± 2.1	14.8 ± 3.7
		Homosexual	14.4 ± 3.2	15.0 ± 2.7	14.2 ± 2.8	15.5 ± 2.7	14.8 ± 1.9	12.9 ± 4.1*
		Bisexual	15.6 ± 3.2	15.5 ± 3.0	13.8 ± 1.8	15.6 ± 3.1	14.5 ± 2.2	14.5 ± 3.4
		P value	0.598	0.555	0.263	0.956	0.103	0.040
	Marital status							
		Single	14.7 ± 3.2	15.2 ± 2.6	14.0 ± 2.6	15.7 ± 2.7	14.7 ± 2.0	13.7 ± 3.9
		Married/stable union	15.1 ± 3.4	15.9 ± 2.1	13.4 ± 2.4	15.5 ± 2.6	13.7 ± 2.3^†^	15.0 ± 3.8
		Divorced	13.9 ± 2.6	14.5 ± 2.7	13.4 ± 2.4	14.2 ± 3.4	14.3 ± 1.1	13.7 ± 3.5
		P value	0.525	0.154	0.379	0.300	0.040	0.229
	Religion							
		Catholic	14.6 ± 3.1	15.0 ± 2.5	13.6 ± 2.9	15.5 ± 2.9	14.3 ± 2.1	13.6 ± 3.6
		Other	14.3 ± 3.3	15.8 ± 2.3	13.6 ± 2.7	15.2 ± 2.6	14.5 ± 2.1	14.5 ± 4.3
		No religion	18.0 ± 2.5^‡^	17.2 ± 1.6^§^	16.0 ± 2.0^||^	17.1 ± 1.6	15.7 ± 1.5	16.6 ± 4.4
		P value	0.003	0.013	0.049	0.137	0.123	0.056
	Schooling							
		Illiterate	14.4 ± 3.7	15.6 ± 1.7	14.0 ± 2.9	15.0 ± 2.5	14.0 ± 1.6	15.4 ± 3.8
		Middle school	14.0 ± 2.7	15.3 ± 2.2	13.2 ± 2.7	15.0 ± 2.5	14.2 ± 2.2	13.4 ± 3.7
		High school	15.4 ± 3.0	16.0 ± 2.1	13.4 ± 2.6	16.1 ± 2.5	14.3 ± 2.0	15.1 ± 4.0
		Bachelor’s degree	14.9 ± 3.6	14.9 ± 2.9	14.5 ± 3.0	15.6 ± 3.0	14.7 ± 2.0	13.6 ± 3.9
		P value	0.241	0.153	0.315	0.235	0.504	0.05

WHOQOL-HIV bref: World Health Organization Quality of Life instrument-HIV bref. Intergroup analysis performed by the Analysis of Variance (ANOVA). Intragroup analysis performed by Tukey test:

* p=0.037;

† p=0.077;

‡ p=0.005;

§ p=0.017;

|| p=0.038.

Analysis of association between scores obtained for the WHOQOL-HIV bref domains and MSDH's layers 3 and 4 showed that men living with a partner had higher scores on average than the single men in regard to domain V (p=0.049). Men with a formal job scored higher on average on domains II (p=0.045) and III (p=0.023) than those without formal jobs. Those with *per capita* income below one times the minimum wage (p=0.003) and between one and two times the minimum wage (p=0.042) scored lower on average in the domain when compared to individuals with *per capita* income greater than three times the minimum wage (Table 4).

**Table 4 -:** Distribution of men with AIDS according to layers 3 and 4 of the Model of Social Determinants of Health and scores obtained for the domains of the WHOQOL-HIV bref (N=138). Fortaleza, CE, Brazil, 2013

Model of Social Determinants of Health			Scores obtained for the WHOQOL-HIV bref’s domains (Mean ± Standard deviation)					
			I	II	III	IV	V	VI
Layer 3								
	Number of children							
		No children	14.7 ± 3.1	15.2 ± 2.6	14.2 ± 2.7	15.6 ± 2.7	14.5 ± 2.0	13.9 ± 4.0
		1 – 2	15.1 ± 3.4	15.6 ± 2.2	13.3 ± 2.8	15.4 ± 3.1	14.1 ± 2.0	13.9 ± 3.8
		≥ 3	14.5 ± 3.2	15.3 ± 2.6	13.1 ± 3.6	15.3 ± 2.0	14.4 ± 2.4	15.1 ± 3.7
		P value	0.874	0.772	0.261	0.863	0.598	0.547
	Number of household members							
		1 – 2	15.0 ± 3.3	15.2 ± 2.1	13.9 ± 2.7	15.6 ± 2.4	14.4 ± 2.1	14.1 ± 4.0
		3 – 5	14.7 ± 3.3	15.6 ± 2.1	14.0 ± 2.7	15.9 ± 2.4	14.6 ± 2.1	13.8 ± 4.0
		≥ 6	14.1 ± 2.9	14.7 ± 3.0	12.6 ± 3.4	14.0 ± 2.9	13.5 ± 1.9	14.9 ± 3.6
		P value	0.715	0.413	0.218	0.054	0.196	0.597
	Lives with partner							
		Yes	14.6 ± 3.2	15.2 ± 2.6	14.0 ± 2.5	15.5 ± 2.8	14.7 ± 1.9*	13.7 ± 3.9
		No	15.2 ± 3.3	15.8 ± 2.1	13.5 ± 3.3	15.5 ± 2.7	13.9 ± 2.2	14.8 ± 3.7
		P value	0.280	0.155	0.153	0.867	0.049	0.158
Layer 4								
	Occupational situation (whether has a formal job contract)							
		Formal job	15.1 ± 3.2	15.7 ± 2.3^†^	14.2 ± 2.6^‡^	15.2 ± 2.8	16.6 ± 2.0	14.3 ± 4.3
		No formal job	14.0 ± 3.1	14.3 ± 3.0	12.5 ± 3.3	15.3 ± 3.0	13.6 ± 2.4	13.7 ± 3.9
		Other	14.6 ± 3.4	14.9 ± 2.3	13.0 ± 2.9	15.5 ± 2.5	14.2 ± 1.7	13.1 ± 3.4
		P value	0.359	0.045	0.015	0.841	0.142	0.360
	per capita income in minimum wage (MW)							
		< 1 MW	15.7 ± 2.7	15.1 ± 2.1	13.0 ± 2.5	13.8 ± 2.7	12.0 ± 1.4^§^	15.5 ± 4.0
		1 -2 MW	14.2 ± 3.3	15.1 ± 2.7	13.2 ± 2.7	15.5 ± 2.4	14.1 ± 2.2^||^	13.6 ± 3.9
		˃ 3 MW	15.0 ± 3.2	15.6 ± 2.3	14.3 ± 2.9	15.7 ± 2.9	14.9 ± 1.8	14.2 ± 3.9
		P value	0.372	0.232	0.059	0.066	0.001	0.762

WHOQOL-HIV bref: World Health Organization Quality of Life instrument-HIV bref. Intergroup analysis performed according to Analysis of variance (ANOVA). Intragroup analysis using T Test,

* p=0.049; and Tukey Test:

† p=0.045;

‡ p=0.023;

§ p=0.003;

|| p=0.042.

## Discussion

The social determinants of health are important because they predict the proportion of variation in one's health condition, sanitary inequity, and health-related behaviors. The social determination of health is rooted in an ethical foundation that is equity, defined as the absence of unfair differences among population groups so that inequities in health are socially-produced differences^(^ 11 ^)^.

According to MSDH's layer 1, which depicts individual determinants, the age of most individuals was between 30 and 49 years old, revealing national dynamics in which most men infected with AIDS between 2000 and 2011 were in this age range^(^ 1 ^)^. In regard to race, the results were similar to those found by a study conducted in Maranhão, Brazil in which mixed race was prevalent^(^ 15 ^)^. Note that individual determinants are, in general, considered to be non-modifiable determinants^(^ 11 ^)^.

The analysis of layer 2 showed that the category of exposure heterosexual was predominant, which also represents the national context^(^ 1 ^)^. This finding involves the issue of vulnerability in regard to HIV/AIDS, which is linked to a set of individual and behavioral aspects that facilitate one becoming infected, in addition to sociopolitical aspects, which are represented by the commitment displayed by authorities, inter-sector actions and funding^(^ 16 ^)^.

Most men with AIDS were single and Catholic, which also was observed in another study^(^ 4 ^)^. In regard to education, most had a bachelor's degree and *per capita* income greater than two times the minimum wage, differing from the findings of other studies, where most men with AIDS had low educational levels and income, and performed occasional jobs without formal contracts^(^ 4 ^,^ 14 ^,^ 17 ^)^. Most men had no children, which may be explained by the considerable number of homosexual (39.9%) and bisexual (13.7%) individuals.

The average scores obtained for the WHOQOL-HIV bref domains were similar to those found in other studies addressing people living with AIDS, highlighting an intermediate perception of QoL^(^ 13 ^-^ 14 ^)^. Note that the lowest scores are for domain III, which represents level of independence. Hence, the individuals face changes in their mobility, activities of daily living, depend on medication and treatment, in addition to experiencing interference in their capacity to work. These results are related to changes in lifestyle after the diagnosis of HIV infection, starting ART, and also as a consequence of stigmatization^(^ 2 ^-^ 3 ^,^ 9 ^,^ 13 ^-^ 14 ^)^.

In the context of MSDH, homosexual men obtained low average scores in regard to spirituality (p=0.037), the domain that refers to forgiveness and blame, concerns over the future, death and dying. This finding reflects the early days of HIV/AIDS, during which the infection was attributed to risk groups, especially homosexual individuals, in addition to the fact that infection meant death, a situation which was later changed due to the advent of ART, which led to increased survival rates for people with AIDS^(^ 8 ^-^ 11 ^)^.

Married men scored lower than single men, on average, in regard to domain V (p=0.077), which refers to physical safety, housing, finance, access to health and social care, the ability to acquire information and learn new skills, leisure, environment and transport. This result may be explained by the fact that married men have to provide for their families, which decreases the *per capita* income of those living in the same household^(^ 4 ^)^.

Those who did not profess a religion scored higher than Catholics in domains I (p=0.005), II (p=0.017) and III (p=0.038). This finding contrasts with related literature, which shows positive correlation between health and spirituality as having a religion supports and strengthens the individual in the face of the adversities imposed by pathological conditions. Additionally, AIDS represents, in the social sphere, a series of disparaging metaphors such as associations with punishment, which would amount to being convicted for socially disapproved behaviors^(^ 18 ^-^ 19 ^)^.

Living with a partner also increased the perception of QoL in regard to domain V (p=0.049). This aspect may be related to social support, which indicates the influence of social interactions with the well-being and health of people. Due to stigma, however, a seropositive status for HIV may hinder social support specifically related to the disease^(^ 20 ^)^.

Having a formal job increased the average scores obtained for domains II (p=0.045) and III (p=0.023), while *per capita* income greater than three times the minimum wage increased the average values of scores in regard to domain V when compared to people's *per capita* income of below one times the minimum wage (p=0.003) and between one and two times the minimum wage (p=0.042).

Given the previous discussion, the social inclusion of people with HIV in the work environment has a considerable positive impact on QoL. What is observed, however, is discrimination both on the part of employers and co-workers. This fact culminates in denying to these people the most elementary right, the right to work. Since the 1990s in Brazil, an inter-ministerial decree prohibits, in the sphere of federal public service, the requirement of HIV tests in pre-hiring exams and in periodic health exams, because being HIV-positive does not cause loss of working capacity and social and/or professional contact with these individuals does not constitute a risk of infection^(^ 21 ^)^.

As for layer 5, we have the distal or macro-determinants, represented by society's economic, cultural, and environmental conditions^(^ 12 ^)^. This layer was not included in the data collection, which may be seen as a limitation of this study. The lack of inclusion was because the study's participants do not recognize the more general macro-determinants or aspects of the society to which they belong, including understanding of health policies directed to the prevention, control, and treatment of HIV/AIDS infection.

In this context, initially, due to a lack of a public policy structured by the State aiming to fight HIV/AIDS, a large number of Non-Governmental Organizations (ONGs) emerged in the country to fight the disease. This coping policy gained momentum by reaching various social groups, when civil society mobilized and ensured its priority, political pressure to accomplish it and funding for it. Currently, Brazil has one of the most progressive policies to cope with HIV/AIDS and is a point of reference for the world^(^ 22 ^)^.

The current policy concerning the prevention, treatment and control of HIV/AIDS is a result of the epidemic's social and historical construction. The first discussions concerning its control were directed to the identification of risk factors, a concept that is no longer used because it resulted in the stigmatization of certain groups, such as homosexuals. Later, it was replaced by the concept of risk behavior, which blamed the population for failures in preventing HIV/AIDS. Hence, these approaches were inefficient and did not favor coping with the epidemic, for they did not consider social, cultural and contextual determinants^(^ 23 ^)^.

The epidemic in Brazil is currently stable and the concept of vulnerability began to be considered in the context of HIV/AIDS, since the epidemic is centered in vulnerable population subgroups^(^ 3 ^,^ 18 ^)^. This means that, in addition to groups initially affected by HIV/AIDS, such as homosexuals, sex workers, intravenous drug users and hemophiliacs, people in stable relationships with a risk behavior and who do not use condoms, adolescents, elderly individuals, those with a low level of education, low income, and living in the interior of the country are also taken into account^(^ 3 ^,^ 18 ^,^ 22 ^-^ 23 ^)^.

## Conclusion

The use of MSDH and the application of the WHOQOL-HIV bref enabled identifying the perceptions of QoL held by men with AIDS, who for the most part considered their QoL to be intermediate. This finding shows that despite the existence of policies concerning access to ART and to healthcare provided in the public health network, other aspects such as social determinants of health should be considered for the populations into which HIV has been disseminated.

Studies of this nature are important to understanding the context in which individuals with HIV/AIDS are inserted because the lives of these individuals can be seen from a multidimensional perspective and interventions can thereby be more efficient. Statistical associations concerning category of exposure, marital status, religion, job contract, and income were observed, confirming the importance of social determinants of health in impacting the way men experience the process of health and disease.

We stress the importance of this study for understanding the context of those living with AIDS, since it can contribute to the delivery of care. Note that further studies are needed to contribute to the QoL of these individuals and reduce morbidity and mortality. Hence, even through there is no cure, HIV infection and AIDS can effectively be considered chronic conditions.
